# Novel approach of dermatophytosis eradication in shelters: effect of *Pythium oligandrum* on *Microsporum canis* in FIV or FeLV positive cats

**DOI:** 10.1186/s12917-021-03001-w

**Published:** 2021-09-01

**Authors:** Martina Načeradská, Michaela Fridrichová, Martina Frühauf Kolářová, Tereza Krejčová

**Affiliations:** 1 Department of Veterinary Sciences, Faculty of Agrobiology, Natural and Food Resources, Czech University of Life Sciences in Prague, Kamýcká 129, 165 21 Prague 6, Czech Republic; 2 Department of Inorganic Chemistry, Faculty of Science, Charles University, Hlavova 8, 128 43 Prague 2, Czech Republic

**Keywords:** Dermatophytosis, FeLV, FIV, Itraconazole, *Microsporum canis*, *Pythium oligandrum*, Animal shelter

## Abstract

**Background:**

Shelters and similar facilities with a high concentration and fluctuation of animals often have problems with various infections, which are usually difficult to solve in such environments and are very expensive to treat. This study investigated the eradication of *Microsporum canis*, the widespread cause of zoonotic dermatophytosis in shelters, even in immunosuppressed feline leukaemia virus or feline immunodeficiency virus positive cats.

**Results:**

Our study showed the increased effectiveness of an alternative topical therapy for affected animals using the mycoparasitic fungus *Pythium oligandrum*, which is gentler and cheaper than the standard systemic treatment with itraconazole, and which can also be easily used as a preventative treatment. A decrease in the number of *M. canis* colonies was observed in cats treated with a preparation containing *P. oligandrum* 2 weeks after the start of therapy (2 cats with P-1 score, 2 cats with P-2 score, 5 cats with P-3 score) compared with the beginning of the study (9 cats with P-3 score = massive infection). The alternative topical therapy with a preparation containing *P. oligandrum* was significantly more effective compared with the commonly used systemic treatment using itraconazole 5 mg/kg in a 6-week pulse. After 16 weeks of application of the alternative topical therapy, the clinical signs of dermatophytosis were eliminated throughout the whole shelter.

**Conclusion:**

The complete elimination of the clinical signs of dermatophytosis in all cats indicates that this therapy will be useful for the management and prevention of zoonotic dermatophytosis in animal shelters.

**Supplementary Information:**

The online version contains supplementary material available at 10.1186/s12917-021-03001-w.

## Background

Dermatophytosis, one of the most widespread zoonoses, is a fungal infection affecting the surface layers of the skin and nails in humans as well as hair and claws in animals [1–5]. Its elimination is difficult and very expensive, especially in large farms or shelters with a high number and fluctuation of animals. This disease is caused by more than 30 different species, especially *Microsporum canis, M. gypseum,* and *Trichophyton mentagrophytes* in animals [3].

Cats are primarily affected by *M. canis*. The typical clinical symptoms are regular and circular alopecia, with hair breakage, desquamation, and sometimes an erythematous margin with a central healing zone. Secondary bacterial infections occur very often, especially in immunosuppressed animals [6]. This dermatophyte can also cause subclinical infections, where infected individuals, especially long-haired cats living in a contaminated environment, termed asymptomatic carriers, might not present with clinical symptoms [7]. Predisposing factors include immunosuppression caused by disease or immunosuppressive treatment, other diseases, nutritional deficits (especially proteins and vitamin A), high temperature, and high humidity [8–10]. In healthy cats, dermatophytosis is a self-limiting disease where the clinical symptoms usually disappear within 4 months if the infection is mild and its source is removed [11, 12]. Shelters are often inhabited with sick, immunocompromised animals that are unable to cope with the infection on their own, and thus such animals often remain in shelters for a long time because they are difficult to adopt. Examples of cat-related infections include feline leukemia virus (FeLV) and feline immunodeficiency virus (FIV). These are serious, incurable retroviral diseases that can be easily transmitted between cats [13–15]. Country recommendations for shelters regarding the handling of FIV and FeLV positive cats are different. Some countries favour euthanasia [16]; however, there is a no-kill policy in the Czech Republic, where there is an effort to place sick animals in suitable conditions. FIV and FeLV positive cats in shelters have a very weakened immune system and they find it difficult to cope with dermatophytosis and therefore require therapy [17, 18]. The treatment of dermatophytosis is recommended for all affected animals (not only FIV and FeLV positive cats) to shorten the disease course and reduce the risk of spreading the infection [12].

Therapy of dermatophyte infections should include a combination of topical and systemic antifungal medications as well as environmental decontamination [3]. Many active substances can be used for systemic therapy (allylamines, azoles, echinocandins, polyenes, griseofulvin, lufenuron), but these substances also cause relatively serious side effects including teratogenicity, embryotoxicity, liver toxicity, anorexia, vomiting, and diarrhoea [6]. Currently, the use of itraconazole in cats is preferred for the systemic treatment of dermatophytosis, despite its relatively high cost. Itraconazole is better tolerated by cats than ketoconazole or griseofulvin, but it also causes side effects such as hypersalivation, anorexia, vomiting, and hepatotoxicity. Although its teratogenicity and embryotoxicity are lower than ketoconazole, it is still contraindicated for pregnant females, animals less than 6 months of age, or animals with liver or kidney disease [3, 6, 19, 20]. Its usual dose is 5 mg/kg by pulse therapy for 4–6 weeks (1 week on, 1 week off) [12, 21], this course of therapy is a registered way of use of itraconazole (Itrafungol, Elanco, Greenfield, USA). Itraconazole is a first generation triazole that works by inhibiting fungal cytochrome P450 enzyme 14α demethylase to prevent the conversion of lanosterol to ergosterol, which maintains cell wall integrity and activity [22]. Solutions and shampoos with active substances (lime sulfur, enilconazole, miconazole, chlorhexidine) and their combinations can be used for topical therapy. Due to the nature of these substances, it is necessary to adhere to the prescribed concentrations, prevent possible contact with the cat’s mucous membranes during their application, and to prevent possible ingestion by the cat, which can cause severe side effects [23–25]. Standard systemic and topical therapies are relatively expensive, require a long duration of application, and can cause side effects in the affected animals because of the nature of their active ingredients. In recent years, the mycoparasitic fungus *Pythium oligandrum* has been used for the topical treatment of dermatophytosis in humans [26] as well as veterinary medicine [27] and is non-pathogenic for animals [27, 28] and humans [26]. It is a non-pathogenic soil oomycete that colonises the root system of many plant species [29]. *P. oligandrum* shows strong mycoparasitism against more than 50 species of fungi and oomycetes. It is commonly used in plant protection, for example against *Fusarium spp., P. spinosum, P. nunn, P. ultimum,* and *P. irregulare* [30]. In vitro, it has also been shown to be effective against dermatophytosis agents such as *M. canis, M. gypseum,* and *T. mentagrophytes* [1, 3, 26]. *P. oligandrum* produces fast-growing hyphae, round oogonies with spikes, and a large number of enzymes (chitinases, cellulases, proteases, and glucanases) collectively referred to as oligandrin, which breaks down the cell wall of host cells leading to complete cytoplasmic destruction and host death [29, 31–33].

Although a combination of topical and systemic therapy is currently recommended for the treatment of dermatophytosis [3, 12], this study focused on the use of gentle topical therapy, which is not otherwise burdensome for cats. The effectiveness of topical therapy using *P. oligandrum* was compared with the recommended and standard systemic itraconazole therapy by pulsed administration for 6 weeks, according to a previous study [12]. In this study, the experimental scheme reported by Puls et al. [12] was followed and adapted for the topical application of the preparation containing *P. oligandrum*. To demonstrate the effect of our proposed therapy, we used immunosuppressed cats (FeLV or FIV positive) of different ages (young to very old), which were not expected to be spontaneously cured.

## Methods

This pilot study was conducted in collaboration with a private asylum for animals in need in the Tibet Shelter (Marefy 44, Bučovice, Czech Republic). The study design, planned sampling, and clinical examination methodology, as well as the proposed treatment protocols, were approved in advance by the owners of the Tibet Shelter and by the Animal Ethics Committee of the Czech University of Life Sciences in Prague. The presented study fully complies with the legislative regulations of the Czech Republic and the Act on the Protection of Animals against Cruelty No. 246/1992 Coll.

### Animals and shelter facility

A shelter with well-adjusted animal management was selected for this study. All animals underwent veterinary examination on admission. Cats placed in a modern quarantine area with fully disinfectable cages were dewormed, vaccinated (vaccine Purevax RCP, Merial, Saint Priest, France, according to the scheme recommended by the manufacturer), neutered, and tested for retroviral infections (FIV and FeLV). Antigen Rapid FIV Ab/FeLV Ag Test Kit (Bionote, Gyeonggi-do, South Korea; Sensitivity: FIV 96.8%, FeLV 94.7%, Specificity: FIV 99.6%, FeLV 99.7%) was used for testing of the cats. FeLV antigen test positive cats were verified by PCR in the Idexx laboratory for FeLV proviral DNA and all of them tested positive, further named as “FeLV positive”. FIV antibody test positive cats were repeatedly tested by the same method, further mentioned as “FIV positive”. Cats not included in the selected group were offered for adoption.

**Scheme 1:**
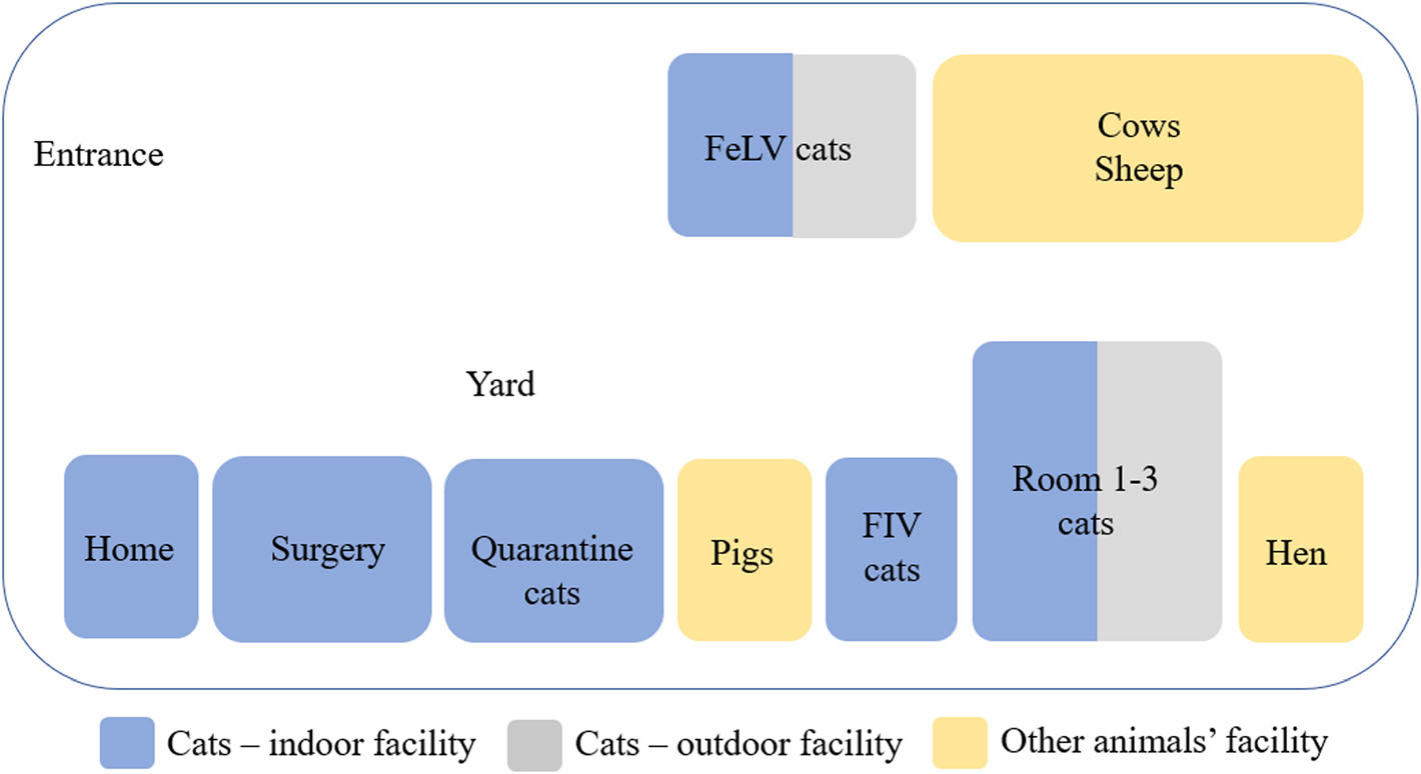
Plan of the shelter

The cats were kept in small groups housed separately in this shelter. Most groups had access to separate outdoor runs. Housing facilities were enriched with play elements, places suitable for climbing, as well as suitable beds and cat trees. The shelter premises were regularly cleaned and disinfected. A plan of the shelter is shown in Scheme 1.

At the beginning of the study, there were 111 cats, 13 dogs, 2 cows, 3 pigs, 6 sheep, 10 hens, 1 rabbit, and 2 guinea pigs, housed in the shelter and cared for by the owners and volunteers.

### Experimental design

#### Part 1

Initial epidemiological screening of all animals present in the shelter to determine the overall prevalence of dermatophytosis and to assess potential contamination in the living environment.

#### Part 2

Study comparing the effectiveness of topical therapy with *P. oligandrum* and systemic itraconazole therapy in FeLV or FIV positive cats. This part lasted 6 weeks and followed a previously reported methodology [12]. During this time, other animals in the shelter, in addition to cats, were treated with a preparation containing *P. oligandrum* to prevent further transmission of dermatophytes between animals in the shelter.

#### Part 3

**Scheme 2:**
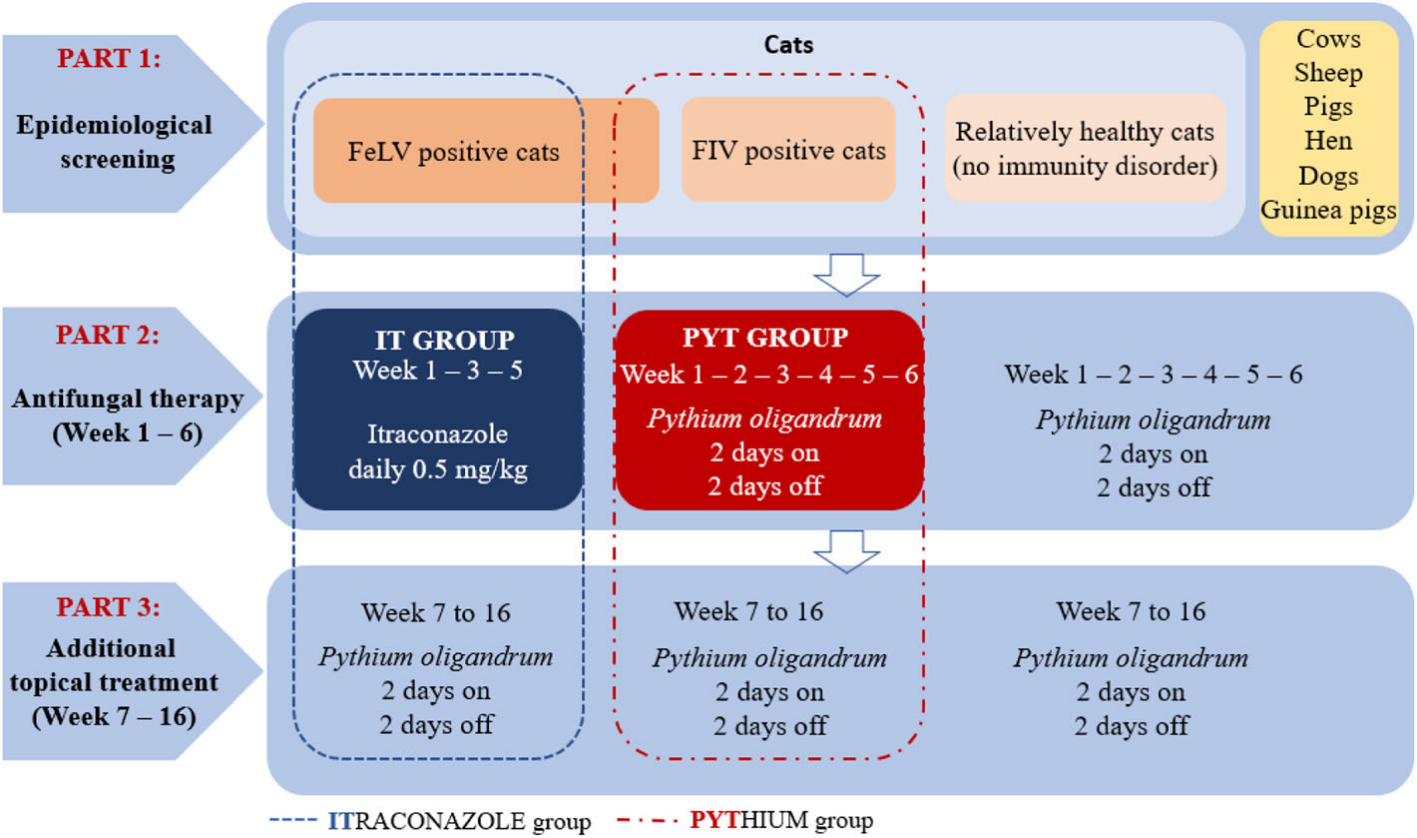
Study flow in the shelter

Continuation of topical therapy with *P. oligandrum* in all sheltered animals for 16 weeks (4 months). Otherwise healthy cats were able to recover on their own if the source of infection was removed. Further design of permanent measures for the management of the shelter and subsequent follow-up was performed. The whole study flow is summarized in Scheme 2.

#### Sampling method (Mackenzie method)

At the beginning and end of the study (after 16 weeks), the full screening of all animals was performed to detect dermatophytes (epidemiological screening). A sample was taken from all animals in the shelter, in addition to cats, for culture to detect dermatophytes. Sampling was performed from the whole body by combing the hair with a sterile toothbrush [34, 35], which was then immediately placed in a sterile resealable plastic bag with a label and shipped to an accredited diagnostic laboratory (Sevaron, Brno, Czech Republic).

Samples were taken in the same way for cats included in the study (groups PYT and IT; more detailed information in Treatment protocols and Scheme 2). Sampling was performed at 2, 4, and 6 weeks. In these groups, samples were taken from affected areas and areas with fluorescence under Wood’s lamp.

#### Fungal cultures

All samples were examined at an accredited diagnostic laboratory (Sevaron s.r.o., Brno, Czech Republic). Dermatophyte Test Medium (DTM) (OXOID CZ; Thermo Fisher Scientific, Brno, Czech Republic) was used to culture the samples. Samples were incubated at 25–29 °C for 14 days. The results were evaluated under a microscope by an experienced mycologist. The results were interpreted using the P-score system: P-0 = negative, P-1 = 1–4 colony forming units (cfu)/plate, P-2 = 5–9 cfu/plate, and P-3 = ≥10 cfu/plate.

#### Diagnostic criteria for dermatophytosis

Positive cats were those with positive clinical examination with use of scoring system [12, 36], Wood’s lamp fluorescence and positive mycological culture (P-score P1 and higher).

#### Treatment protocols

Regarding the very gentle approach to cats, their location, and the statistical evaluation of the study results, FeLV or FIV positive cats with a positive finding of dermatophytes were selected and divided into 2 groups that received different treatments:

##### IT group

10 FeLV positive cats that were easy to handle (see Supplementary data 1). This group of cats was treated by the oral administration of itraconazole (Itrafungol, Elanco) according to the schedule: week of treatment (1x daily administration of the drug, at a dose of 5 mg/kg), 1 week break (all repeated 3 times) as previously reported [12, 37].

##### PYT group

9 FIV or FeLV positive cats (see Supplementary data 1). This group of cats was treated with a preparation containing *P. oligandrum* Ecosin (BARD s.r.o., Prague, Czech Republic) with the solution applied directly to the fur (three effervescent tablets were diluted in six litres of lukewarm water per one application for the whole facility). Dermasin oil (BARD s.r.o., Prague, Czech Republic) was applied to the affected areas of the head on each cat (cca 1 ml pec cat). *P. oligandrum* was applied according to the Scheme 2: 2 days application of the solution to the fur, 2 days break (for a total of 6 weeks). The owner of the shelter applied the product gently, by stroking the cats with a glove soaked in the product or by applying the oil directly to the affected areas of the head.

All animals that were not included in the selected groups were treated in the same way as the PYT group during the study to treat their symptoms and prevent the spread of dermatophytosis among the animals in the shelter. Substances soaked in the product, which were placed in the entrance to the outdoor areas, were also used to apply the product containing *P. oligandrum* (Ecosin, BARD). In this way, cats were in contact with the product when passing through the entrance, including non-socialized cats for which normal handling was impossible. Therefore, timid cats did not have to be bathed in the product, which is usually a very stressful procedure.

### Examination of cats

Every 14 days, swabs were taken from the fur of cats in both groups to examine the presence of dermatophytes.

At the beginning (day 0) and end (after 6 weeks) of the study, biochemical analysis of blood was performed for all cats in the IT and PYT groups (Idexx Catalyst one analyser) (see Supplementary data 2).

At all times, the cats were under veterinary supervision and their health was regularly checked, using a method to minimize stress.

### Environmental cleaning

Extensive cleaning of the shelter was performed before the start of the study. All areas, including the walls, were washed and treated with disinfectant. Incidin plus (1%) (Ecolab s.r.o., Prague, Czech Republic), which has no antifungal properties, was used as a disinfectant. This disinfection was used throughout the study and was alternated with Incidin OxyDes (1%) (Ecolab s.r.o., Prague, Czech Republic).

To reduce the fungal contamination of the environment, the areas where the animals were kept were also regularly treated during the study: 1x weekly application of a solution with *P. oligandrum* in a preparation intended for surface treatment (Biorepel, Biopreparáty s.r.o., Prague, Czech Republic). A detailed breakdown of cleaning is provided in Supplementary data 3.

### Statistical analysis

The method of least squares – linear model GLM with fixed effects at a level of significance α ≥ 0.05 (SAS software) was used to demonstrate statistically significant differences.

The model equation for the calculation was: Y = type of treatment + weeks of treatment + type of treatment * weeks of treatment + e.

Y – is a manifestation (P – score), type of treatment – fixed effect (pythium, itraconazole), weeks of treatment – fixed effect (0, 2, 4, and 6 weeks). A table providing information on the evidence of effects (independent variables) on disease manifestation (dependent variables) is provided in Supplementary data 4.

## Results

### Part 1

The results of the initial screening of all animals present in the shelter confirmed a massive dermatophyte infection in 47/111 (42%) cats, 3/13 (23%) dogs, and 2/2 (100%) guinea pigs. The other animals were negative (see Supplementary data 5 and 6). Scoring system for lesions and Wood’s lamp examination was adjusted according to the literature [12, 36]. This screening also showed a significantly greater detection of dermatophyte fungi in cats with retroviral infection: 100% of FIV positive cats (8/8) and 50% of FeLV positive cats (14/28) were affected compared with other more or less healthy cats and cats accepted for quarantine, where the detection of dermatophyte positivity was 33% (25/75).

### Part 2

**Fig. 1:**
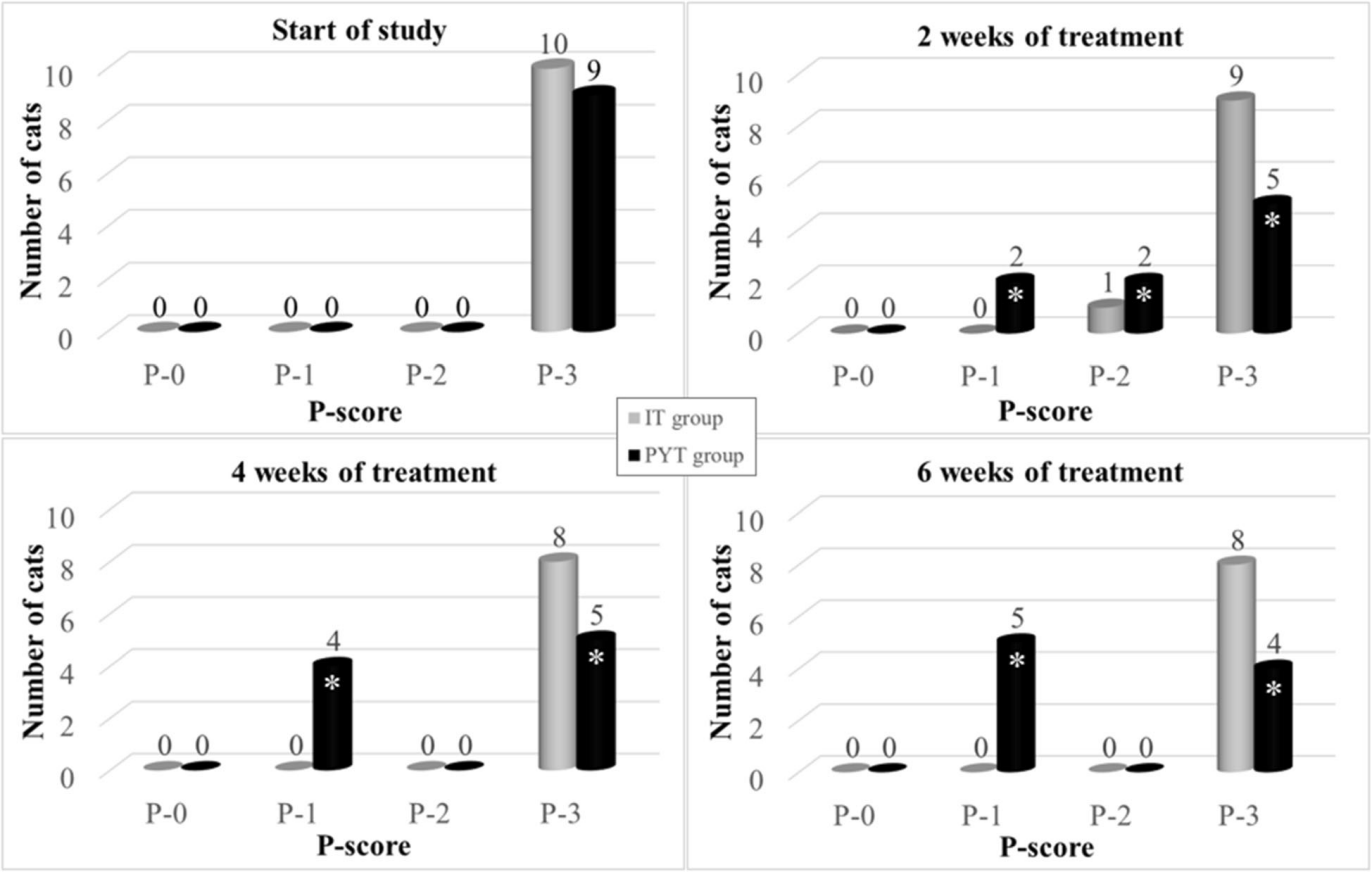
Graphs comparing the two treatments for dermatophytosis at the beginning of the study, and after 2, 4, and 6 weeks of treatment. Cats in the IT group were treated with standard itraconazole and cats in the PYT group were treated with topical *P. oligandrum* (see Materials and Methods, Treatment protocols). *Significantly more effective therapy evaluated with linear GLM model is marked with asterisk (see Methods, Statistical analysis)

In cats, the dominant cause of dermatophytosis is *M. canis*. In our study, the cultivation of samples taken from areas positive by Wood’s lamp examination, confirmed a massive infection (i.e., ≥10 cfu) of *M. canis* in all cats from the IT and PYT groups. After the first 2 weeks of treatment, there was a significant decrease in the number of *M. canis* cfu in the PYT group compared with the IT group. Regarding P-scores, in the PYT group, 2 cats were scored as P-1, 2 as P-2, and 5 cats as P-3. In the IT group, all cats were scored as P-3 (see Fig. 1) and they showed high levels of salivation and loss of appetite after the administration of itraconazole. Furthermore, these cats began to refuse this preparation and their treatment was more difficult. No adverse reactions were observed in the group of cats treated with topical *P. oligandrum*. This was due to the nature of the active substance and the way it was applied: the cats were not subjected to any potentially stressful manipulations during its application.

After 4 weeks of treatment, a course similar to that after 2 weeks of treatment was observed (see Fig. 1). In the IT group, all cats were scored as P-3. Two cats died one of which was diagnosed with feline infectious peritonitis, FIP (post-mortem autopsy and ascitic fluid was coronavirus PCR-positive). Autopsy of the second cat revealed a rupture of the liver and a diagnosis of amyloidosis was confirmed by histopathology examination. Four weeks after the administration of itraconazole, the enormous salivation and loss of appetite remained in cats in the IT group, they began to show signs of stressed behaviour before itraconazole administration and their treatment was problematic. In the PYT group, 4 cats were scored as P-1 and 5 as P-3. Of note, the cats were not afraid of the treatment, which was applied by petting with a wet glove, and they showed better socialization by approaching the person of their own free will during the treatment application.

After 6 weeks of treatment, a similar course was still evident. In the IT group, all cats were scored as P-3 whereas in the PYT group, 5 cats were scored as P-1 and 4 cats as P-3 (see Fig. 1).

### Part 3

The study continued for another 10 weeks (16 weeks in total = 4 months). All animals present in the shelter, as well as cats in the original IT group, were treated with *P. oligandrum* topical therapy (see Material and Methods) during this time. Itraconazole systemic therapy was not continued due to the occurrence of adverse reactions.

**Fig. 2:**
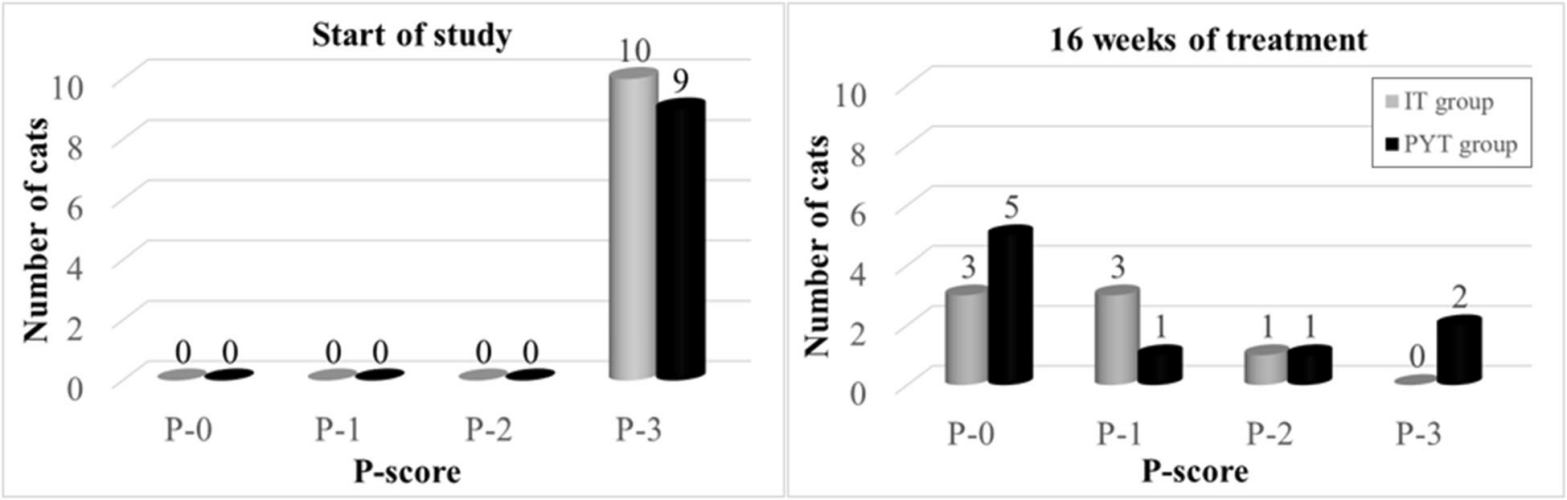
Graphs comparing the two treatments for dermatophytosis at the beginning of the study and after 16 weeks of treatment. Cats in the IT group were treated with standard itraconazole for 6 weeks and then for 10 weeks with topical *P. oligandrum*. Cats in the PYT group were treated with topical *P. oligandrum* (see Materials and Methods, Treatment protocols). Statistically significant differences were observed between the start of the study and after 16 weeks of treatment. No statistically significant differences were observed between the chosen method of therapy after 16 weeks of treatment

After 16 weeks and 10 weeks, respectively, of topical therapy with *P. oligandrum*, 3 cats were scored as negative (P-0), 3 as P-1, and 1 as P-2. One cat died of FIP infection (confirmed by autopsy and PCR examination) in the former IT group. In the PYT group, 5 cats were evaluated as negative (P-0), 1 as P-1, 1 as P-2, and 2 as P-3 (see Fig. 2). Two cats scored as P-3 suffered from another disease. One was a very old FeLV positive blind cat with suspected Conn’s syndrome. The second was an old FeLV positive cat with acute inflammation of the oral cavity, which could not take care of itself and which might have been a source of contamination to other cats. These cats were further treated with topical *P. oligandrum.* When examined by Wood’s lamp after 16 weeks, no FIV or FeLV cats were positive and none showed clinical signs of dermatophytosis (Supplementary data 6 and 7).

**Fig. 3:**
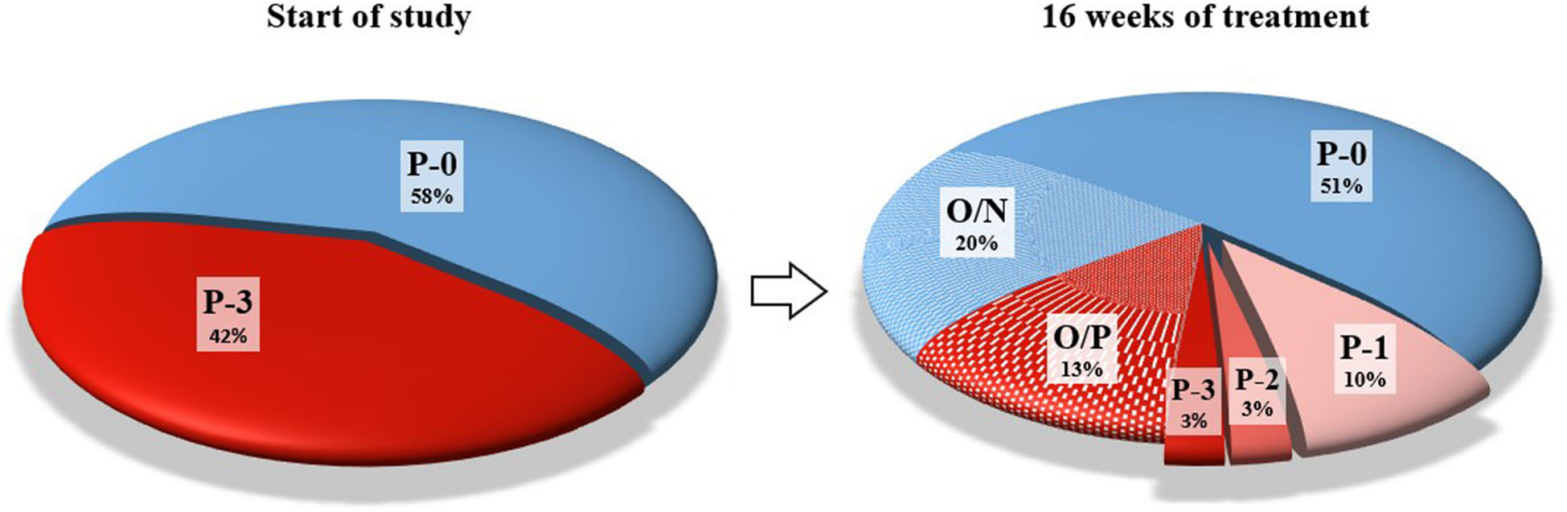
Comparison of the incidence of dermatophytosis in cats at the beginning of the study and after 16 weeks of treatment with topical *P. oligandrum*. P-0: *M. canis* negative, P-1: 1–4 cfu of *M. canis*, P-2: 5–9 cfu of *M. canis*, P-3: ≥10 cfu of *M. canis*, O/N: out of study cats (adopted or death), which were *M. canis* negative at the beginning of the study, O/P: out of study cats (death), which were *M. canis* positive (P-3) at the beginning of the study

After 16 weeks (4 months), a final epidemiological screening of all shelter animals was performed. A significant improvement in the epidemiological situation (see Fig. 3, Supplementary data 5 and 6) in the shelter was observed in terms of the occurrence of dermatophytosis in all animals when compared with the beginning of the study (see Fig. 3). All new animals admitted to the shelter during the study were quarantined, tested for dermatophytes, and prophylactically treated with a topical application of *P. oligandrum*. At the beginning of the study, of 111 cats present in the shelter, 64 (58%) were negative and 47 (42%) were *M. canis* positive with a P-3 score. During the study, 22 (20%) cats that were initially negative were adopted or died, and 15 (14%) cats that were originally positive (P-3), died or had to be euthanised due to poor health (a panleukopenia infection occurred in quarantine cats during the study). After 16 weeks (4 months) of treatment, 17 of the original 111 cats remained positive (16%). Eleven of these positive cats were scored as P-1 (10% of the original 111 cats), which can be considered to be caused by contamination from the environment. These cats did not show clinical signs and were negative by Wood’s lamp examination. Of the other 6 cats (6%), 3 were scored as P-2 and 3 as P-3. These cats had no clinical signs of dermatophytosis but all of them suffered from other acute illnesses at that time. One died due to multiple neoplasia, one had to undergo enucleation of the eye due to massive inflammation, and the other four underwent intensive treatment.

The results of the other tested animal species in the shelter are shown in Supplementary data 5.

**Scheme 3:**
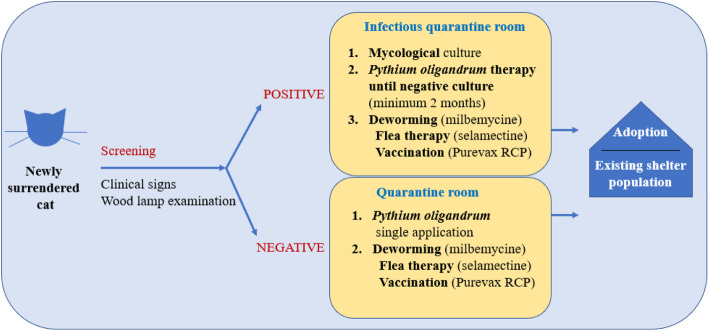
Overview of measures for cats newly admitted to a shelter

As mentioned previously, the important task of the study was setting a sustainable management plan for the shelter in order to prevent new outbreak of the dermatophytosis. At the end of the study, we suggested recommendations summarised in scheme 3. At follow-up 1 year after the start of the study, no clinical signs of dermatophytosis were observed in cats that had stayed in the shelter for a long time. The shelter still follows an established procedure during admission; cats with skin lesions are tested for dermatophytes (fungal culture, Wood’s lamp testing) and prophylactically treated with *P. oligandrum* (see Scheme 3). Shelter spaces are treated at regular intervals.

## Discussion

In this study, we verified a gentle and inexpensive procedure for the eradication of dermatophytosis in animals, targeted at the needs of shelters, asylums, or large animal farms, using the mycoparasitic fungus *P. oligandrum*. Our study was based on a previous report showing the beneficial effect of the mycoparasitic fungus *P. oligandrum* on *M. canis* [1, 27], one of the most common causes of dermatophytosis in cats and dogs.

The results of the initial epidemiological screening showed a relatively high overall prevalence of dermatophytosis in cats in our shelter. A total of 42% of cats were positive, despite the quality of the management of the shelter. Of these cats, 23% of otherwise healthy cats were dermatophytosis positive, which is about 10 times higher than previously reported for other countries (2.4% USA, 3.6% Canada, 1.3% UK) [3].

As data on the average prevalence of dermatophytosis in the Czech Republic have not been published, it is not possible to objectively assess the cause of such a high prevalence in the monitored shelter. In our opinion, the environmental load of dermatophytes in this locality might have a significant role on the results of this study. The massive occurrence of *M. canis* and other common keratinophilic fungi species comparable to the occurrence of dermatophytes in similar studies, was confirmed here [38, 39].

After 2 weeks of the recommended therapy, a statistically significant difference was seen between the two treatment groups. Our topical therapy with *P. oligandrum* had a statistically significant beneficial effect compared with the standard systemic therapy using itraconazole. A similar effect was seen after 4 and 6 weeks of therapy. In a group of cats treated with standard itraconazole, side effects potentially related to this preparation were observed [6]. Puls et al. [12] reported the occurrence of hypersalivation and a slightly increased frequency of anorexia and vomiting in cats, but did not describe behavioural changes in cats treated with itraconazole. In our study, we observed changes in the behaviour of cats. With an increasing duration of itraconazole administration, cats showed signs of stressful behaviour, fear of its application, and aggression. Two cats treated with itraconazole died during the 6 weeks of therapy. Although it is not possible to prove a causal relationship between these deaths and the active substance used (itraconazole), we concluded that the increase in stress to which cats were exposed during this application may have significantly contributed to the acute phase of both diseases and the death of the cats.

Puls et al. [12] described the rapid onset of action of itraconazole. By the second week of treatment, they observed a clinical cure and after 4 weeks they observed a mycological cure (2 consecutive negative cultures). In our study, itraconazole therapy outcome was different. After 6 weeks of therapy, none of the groups was completely mycologically cured of dermatophytosis, but 1 cat out of 8 from IT group and 5 cats out of 9 from PYT group did not exhibit any clinical signs (supplementary data 6 and 7). Six weeks is the standard recommended duration of treatment with itraconazole in otherwise healthy cats [3, 12]. Previous studies usually exclude immunosuppressed cats from the shelter population prior to treatment for dermatophytosis [40]. These cats were intentionally included in our study. To date, the higher incidence of fungal diseases in retrovirus-affected cats has not been clearly established [3, 39]; however, in our study, there was a significant difference in the incidence of dermatophytosis between sick cats (FIV 100%, FeLV 52%) and healthy cats (23%). A previous study reported a similar finding, where dermatophytosis was observed in 74% of FIV positive cats and in 25% of FIV seronegative cats from shelters and households with access outside [34].

When comparing the effect of both therapies, after 2 weeks a statistically significant decrease in the number of *M. canis* cfu was evident in cats treated with topical *P. oligandrum* compared with systemic itraconazole. Of note, the topical therapy had a faster treatment onset and cats with declining *M. canis* cfu were potentially much less dangerous in terms of transmitting and spreading this infection to other cats, animals, and humans with whom they came into contact.

After 16 weeks of therapy, there was no statistically significant difference in P-scores between the IT and PYT groups. Of note, dermatophytosis was almost eradicated in both groups (cats exclusively receiving topical *P. oligandrum* and cats receiving systemic therapy with itraconazole followed by topical *P. oligandrum*) in the shelter. Apart from 3 cats that had a poor clinical condition requiring intensive treatment, the other cats were cured of dermatophytosis despite them being FeLV and FIV positive and very old. Of the original 42% of positive cats, at the end of the study 3% of cats were scored as P-3 (clinically very sick requiring intensive treatment), 3% were scored as P-2, and 10% were scored as P-1. These results might be partly related to contamination from the environment (see Fig. 3).

## Conclusion

In this study, we demonstrated the comparable efficacy of an alternative to itraconazole for dermatophytosis treatment using the topical application of a product containing *P. oligandrum*. Of note, the alternative treatment with *P. oligandrum* had a faster beneficial effect compared with itraconazole. Therefore, this topical therapy is recommended as a cheaper and gentler alternative to itraconazole for the treatment and prevention of dermatophytosis, especially in animal shelters or large farms, where the classic method of therapy is expensive and difficult to manage, even in ill animals (FeLV or FIV positive).

## Supplementary Information

**Additional file 1.**

## Data Availability

Detailed data available in attached Supplementary file.

## Abbreviations

FeLV: feline leukaemia virus
FIV: feline immunodeficiency virus
DTM: dermatophyte test medium
cfu: colony forming units
