# Supplementary material for: Novel approach of dermatophytosis eradication in shelters: effect of Pythium oligandrum on Microsporum canis in FIV or FeLV positive cats
Source: BMC Vet Res. 2021 Sep 1;17:290. doi: 10.1186/s12917-021-03001-w (PMC8409471; doi:10.1186/s12917-021-03001-w)

**Supplementary data**

**Novel approach of dermatophytosis eradication in shelters: Effect of *Pythium oligandrum* on *Microsporum canis* in FIV or FeLV positive cats**

Martina Načeradská^1*^, Michaela Fridrichová^2^, Martina Frühauf Kolářová^1^, Tereza Krejčová^1^

^1^ Department of Veterinary Sciences, Faculty of Agrobiology, Natural and Food Resources, Czech University of Life Sciences in Prague, Kamýcká 129, Prague 6, 165 21, Czech Republic

^2^ Department of Inorganic Chemistry, Faculty of Science, Charles University, Hlavova 8, Prague 2, 128 43, Czech Republic

Correspondence: [naceradska@af.czu.cz](mailto:naceradska@af.czu.cz)

Supplementary data 1. Overview of cats included in the compared groups IT and PYT:

| **Cat** | **Age** | **Sex** | **Neutered** | **Vaccination** | **Deworming** | **FIV** | **FeLV** |
| --- | --- | --- | --- | --- | --- | --- | --- |
| IT_01 | 1 | M | + | + | + | - | + |
| IT_02 | 4 | F | + | + | + | - | + |
| IT_03 | 5 | M | + | + | + | - | + |
| IT_04 | 7 | M | + | + | + | - | + |
| IT_05 | 1 | M | - | + | + | - | + |
| IT_06 | 1 | F | + | + | + | - | + |
| IT_07 | 1 | F | + | + | + | - | + |
| IT_08 | 10 | M | + | + | + | - | + |
| IT_09 | 8 | F | + | + | + | - | + |
| IT_10 | 1 | M | - | + | + | - | + |
|  |  |  |  |  |  |  |  |
| PYT_01 | 10 | M | + | + | + | + | - |
| PYT_02 | 6 | M | + | + | + | + | - |
| PYT_03 | 5 | F | + | + | + | + | - |
| PYT_04 | 10 | F | + | + | + | + | - |
| PYT_05 | 1 | M | + | + | + | + | - |
| PYT_06 | 1 | M | + | + | + | - | + |
| PYT_07 | 10 | F | + | + | + | - | + |
| PYT_08 | 17 | F | + | + | + | - | + |
| PYT_09 | 10 | F | + | + | + | - | + |

Supplementary data 2: Results of blood biochemistry in cats included in the compared groups IT and PYT (S0 – at the beginning; S1 – after 6 weeks of treatment).

| **Chemistry** | **Sample** | **GLU** | **CREA** | **UREA** | **BUN/CREA** | **TP** | **ALB** | **GLB** | **ALB/GLB** | **ALT** | **ALP** |
| --- | --- | --- | --- | --- | --- | --- | --- | --- | --- | --- | --- |
| **Reference value** |  | 4,11 - 8,84 mmol/L | 71 - 212 µmol/L | 5,7 - 12,9 mmol/L |  | 57 - 89 g/L | 22 - 40 g/L | 28 - 51 g/L |  | 12 - 130 U/L | 14 - 111 U/L |
| **IT_01** | S0 | 4,87 | 188 | 4,7 | 6 | 87 | 28 | 59 | 0,5 | 67 | 38 |
|  | S1 | 4,84 | 112 | 5,4 | 12 | 83 | 28 | 55 | 0,5 | 48 | 50 |
| **IT_02** | S0 | 4,12 | 130 | 8,1 | 15 | 89 | 30 | 59 | 0,5 | 82 | 43 |
|  | S1 | 6,33 | 105 | 5,5 | 13 | 84 | 27 | 58 | 0,5 | 72 | 45 |
| **IT_03** | S0 | 4,13 | 124 | 7,9 | 16 | 75 | 27 | 49 | 0,5 | 39 | 39 |
|  | S1 | 4,17 | 119 | 7,7 | 16 | 74 | 28 | 46 | 0,6 | 34 | 48 |
| **IT_04** | S0 | 1,56 | 144 | 8,0 | 14 | 77 | 32 | 44 | 0,7 | 71 | 17 |
|  | S1 | 4,54 | 119 | 7,5 | 16 | 74 | 29 | 44 | 0,7 | 54 | 31 |
| **IT_05** | S0 | 1,62 | 97 | 6,0 | 15 | 82 | 31 | 51 | 0,6 | 140 | 37 |
|  | S1 | 4,5 | 75 | 5,6 | 18 | 90 | 34 | 56 | 0,6 | 92 | <10 |
| **IT_06** | S0 | 4,05 | 93 | 5,5 | 15 | 77 | 32 | 44 | 0,7 | 33 | 26 |
|  | S1 | 4,91 | 107 | 5,3 | 12 | 84 | 34 | 50 | 0,7 | 29 | 24 |
| **IT_07** | S0 | 3,91 | 128 | 5,1 | 10 | 83 | 32 | 51 | 0,6 | 62 | 22 |
|  | S1 | 4,57 | 121 | 7,2 | 15 | 81 | 27 | 54 | 0,5 | 22 | 21 |
| **IT_08** | S0 | 2,82 | 146 | 4,7 | 8 | 79 | 32 | 47 | 0,7 | 42 | 64 |
|  | S1 | 5,8 | 137 | 5,9 | 11 | 82 | 32 | 50 | 0,6 | 63 | 69 |
| **IT_09** | S0 | 1,33 | 260 | 12,6 | 12 | 75 | 28 | 46 | 0,6 | 92 | 23 |
|  | S1 |  |  |  |  |  |  |  |  |  |  |
| **IT_10** | S0 | 4,44 | 115 | 7,3 | 16 | 88 | 26 | 62 | 0,4 | 22 | <10 |
|  | S1 |  |  |  |  |  |  |  |  |  |  |

| **Chemistry** | **Sample** | **GLU** | **CREA** | **UREA** | **BUN/CREA** | **TP** | **ALB** | **GLB** | **ALB/GLB** | **ALT** | **ALP** |
| --- | --- | --- | --- | --- | --- | --- | --- | --- | --- | --- | --- |
| **Reference value** |  | 4,11 - 8,84 mmol/L | 71 - 212 µmol/L | 5,7 - 12,9 mmol/L |  | 57 - 89 g/L | 22 - 40 g/L | 28 - 51 g/L |  | 12 - 130 U/L | 14 - 111 U/L |
| **PYT_01** | S0 | 4,79 | 167 | 10,0 | 15 | 85 | 31 | 54 | 0,6 | 25 | 20 |
|  | S1 | 5,12 | 204 | 7,9 | 10 | 89 | 32 | 58 | 0,6 | 22 | 21 |
| **PYT_02** | S0 | 5,52 | 153 | 6,6 | 11 | 81 | 31 | 50 | 0,6 | 35 | 54 |
|  | S1 | 5,39 | 155 | 6,0 | 10 | 79 | 31 | 49 | 0,6 | 75 | 40 |
| **PYT_03** | S0 | 5,37 | 146 | 5,2 | 9 | 85 | 31 | 54 | 0,6 | 72 | 71 |
|  | S1 | 5,84 | 159 | 7,7 | 12 | 93 | 30 | 63 | 0,5 | 25 | 47 |
| **PYT_04** | S0 | 4,6 | 132 | 7,5 | 14 | 79 | 32 | 47 | 0,7 | 105 | 33 |
|  | S1 | 5,6 | 150 | 8,3 | 14 | 86 | 34 | 52 | 0,6 | 87 | 19 |
| **PYT_05** | S0 | 4,83 | 124 | 5,8 | 12 | 80 | 32 | 47 | 0,7 | 46 | 59 |
|  | S1 | 5,4 | 156 | 5,9 | 9 | 81 | 33 | 48 | 0,7 | 45 | 45 |
| **PYT_06** | S0 | 6,3 | 153 | 5,8 | 9 | 95 | 25 | 70 | 0,4 | 92 | <10 |
|  | S1 | 4,03 | 152 | 10,3 | 17 | 79 | 30 | 49 | 0,6 | 95 | 44 |
| **PYT_07** | S0 | 4,66 | 106 | 5,7 | 13 | 100 | 26 | 73 | 0,4 | 20 | 12 |
|  | S1 | 4,93 | 84 | 7,0 | 21 | 85 | 26 | 59 | 0,4 | 15 | 22 |
| **PYT_08** | S0 | 5,11 | 181 | 9,5 | 13 | 71 | 28 | 42 | 0,7 | 48 | 18 |
|  | S1 | 4,32 | 196 | 14,0 | 18 | 68 | 27 | 41 | 0,7 | 52 | 56 |
| **PYT_09** | S0 | 9,23 | 80 | 4,1 | 13 | 64 | 28 | 37 | 0,8 | 13 | 21 |
|  | S1 | 5,41 | 115 | 12,3 | 27 | 75 | 29 | 46 | 0,6 | 51 | 22 |

Supplementary data 3:

Cleaning measures in the shelter:

Every day, all floor areas are swept and wiped, Incidin plus (1%) alternates regularly with Incidin Oxydes (1%).

Once a week, Biorepel is applied to floors and furniture and allowed to dry.

A large cleaning takes place once a month. By large cleaning we mean vacuuming, wiping smooth surfaces with disinfectants and washing and cleaning the furniture, including hard-to-reach areas. Careful mechanical removal of coarse impurities of inorganic and organic origin using a vacuum cleaner with high-quality filters serves to reduce the occurrence of life stages of fungal agents and mites, which can contribute to the spread of these agents. Therefore, all floor surfaces and surfaces of cat scratches and other equipment for cats are first carefully vacuumed. The floor and smooth surfaces, including walls, are then treated with disinfectant. All furnishing textiles, such as bedding and blankets, are replaced with freshly washed and dried to the highest allowable temperatures. After the treated surfaces have dried, the Biorepel solution is applied to the entire environment and allowed to dry.

Supplementary data 4:

Information on the evidence of effects (independent variables) on disease manifestation (dependent variables):

| Effect |  | | | | |
| --- | --- | --- | --- | --- | --- |
|  | SS | df | MS | F | p |
| Intercept | 538.2760 | 1 | 538.2760 | 1143.139 | 0.000000 |
| Therapy | 10.1227 | 1 | 10.1227 | 21.498 | 0.000017 |
| Week | 2.9924 | 3 | 0.9975 | 2.118 | 0.106253 |
| Therapy*Week | 4.7453 | 3 | 1.5818 | 3.359 | 0.023887 |
| Error | 31.0778 | 66 | 0.4709 |  |  |

Supplementary data 5: Results of epidemiological screening in all animals in the shelter at the beginning and the end of the study.

| Start of study |  |  |  |  |  | End of study |  |  |  |  |  |
| --- | --- | --- | --- | --- | --- | --- | --- | --- | --- | --- | --- |
| Species |  |  |  |  |  | Species |  |  |  |  |  |
| cat | 111 |  |  |  |  | cat | 111 |  |  |  |  |
|  |  | negative | 64 |  |  |  |  | negative | 57 |  |  |
|  |  | positive | 47 |  |  |  |  | out of study | 37 |  |  |
|  |  |  |  | P-1 | 0 |  |  | positive | 17 | P-1 | 11 |
|  |  |  |  | P-2 | 0 |  |  |  |  | P-2 | 3 |
|  |  |  |  | P-3 | 47 |  |  |  |  | P-3 | 3 |
| dog | 13 |  |  |  |  | dog | 13 |  |  |  |  |
|  |  | negative | 10 |  |  |  |  | negative | 9 |  |  |
|  |  | positive | 3 |  |  |  |  | out of study | 2 |  |  |
|  |  |  |  | P-1 | 0 |  |  | positive | 2 |  |  |
|  |  |  |  | P-2 | 0 |  |  |  |  |  |  |
|  |  |  |  | P-3 | 3 |  |  |  |  |  |  |
| guinea pig | 2 |  |  |  |  | quinea pig | 2 | negative | 2 |  |  |
|  |  | negative | 0 |  |  |  |  | positive | 0 |  |  |
|  |  | positive | 2 |  |  |  |  |  |  |  |  |
|  |  |  |  | P-1 | 0 |  |  |  |  |  |  |
|  |  |  |  | P-2 | 0 |  |  |  |  |  |  |
|  |  |  |  | P-3 | 2 |  |  |  |  |  |  |
| rabbit | 1 |  |  |  |  | rabbit | 1 |  |  |  |  |
|  |  | negative | 1 |  |  |  |  | negative | 1 |  |  |
|  |  | positive | 0 |  |  |  |  | positive | 0 |  |  |
| pig | 3 |  |  |  |  | pig | 3 |  |  |  |  |
|  |  | negative | 3 |  |  |  |  | negative | 3 |  |  |
|  |  | positive | 0 |  |  |  |  | positive | 0 |  |  |
| cow | 2 |  |  |  |  | cow | 2 |  |  |  |  |
|  |  | negative | 2 |  |  |  |  | negative | 2 |  |  |
|  |  | positive | 0 |  |  |  |  | positive | 0 |  |  |
| sheep | 2 |  |  |  |  | sheep | 2 |  |  |  |  |
|  |  | negative | 2 |  |  |  |  | negative | 2 |  |  |
|  |  | positive | 0 |  |  |  |  | positive | 0 |  |  |
| hen | 2 |  |  |  |  | hen | 2 |  |  |  |  |
|  |  | negative | 2 |  |  |  |  | negative | 2 |  |  |
|  |  | positive | 0 |  |  |  |  | positive | 0 |  |  |
|  |  |  |  |  |  |  |  |  |  |  |  |

Supplementary data 6: Development of clinical signs scoring and P- Scores for IT and PYT group during the study

Supplementary data 7: Graphical comparison of clinical scoring and P-Scores for IT and PYT group during the study
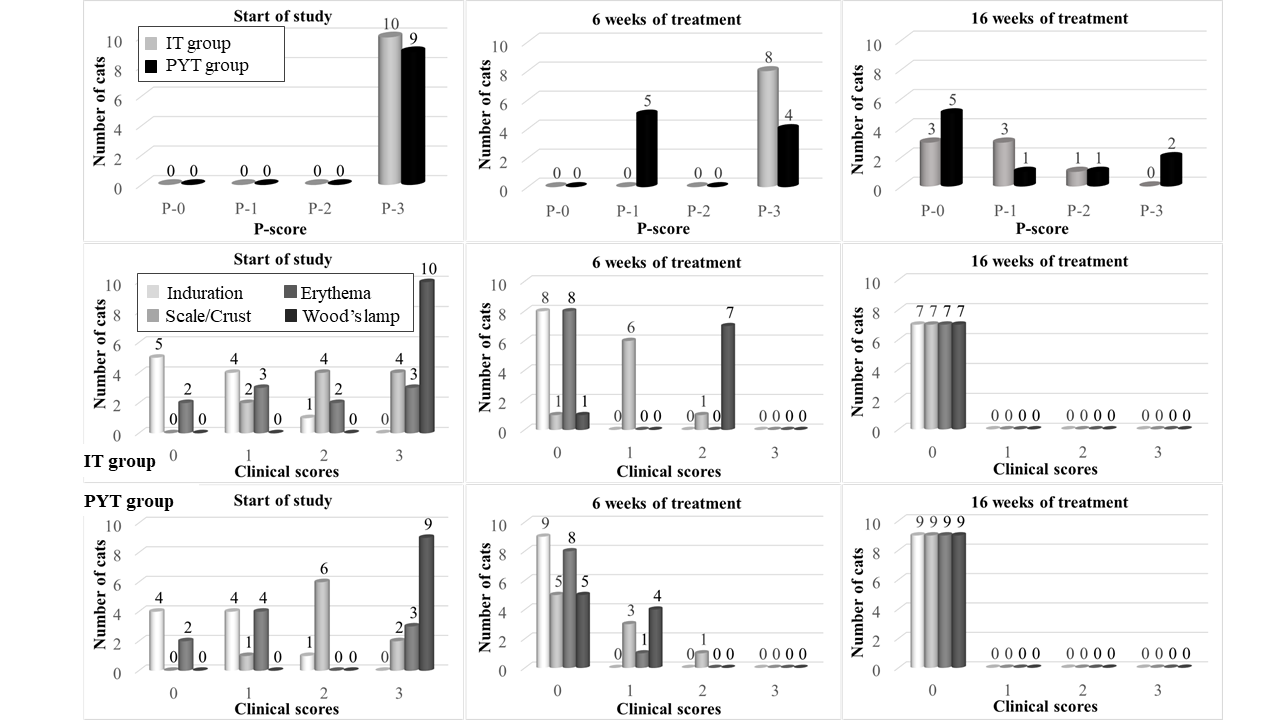

Supplement: Supplementary file 1 — Additional file 1. [file 12917_2021_3001_MOESM1_ESM.docx]
